# Modulation of radiosensitivity of DU145 prostate carcinoma cells by simvastatin

**DOI:** 10.1007/s00432-022-04364-9

**Published:** 2022-09-21

**Authors:** Verena Korte, Guenther Gademann, Ahmed Gawish, Hans-Joachim Ochel

**Affiliations:** 1 Laboratory for Radiation Biology, Clinic for Radiotherapy, Medical Faculty, Otto-von-Guericke-University, Magdeburg, Germany; 2 Radiation Oncology, Universitätsklinikum Magdeburg, Leipziger Str. 44, DE 39120 Magdeburg, Germany

**Keywords:** Simvastatin, DU145, Radiosensitivity, Prostate cancer, HMG-CoA-reductase

## Abstract

**Purpose:**

To investigate antiproliferative effects of simvastatin in combination with ionizing radiation on DU145 prostate cancer cells and its influence on cellular HMG-CoA-reductase levels.

**Methods:**

Proliferative responses of DU145 cells were estimated by means of a clonogenic assay or the crystal violet procedure. HMG-CoA-reductase levels were measured by western blot analysis.

**Results:**

The antiproliferative effects of simvastatin and radiation are dependent on simvastatin dose, radiation dose and treatment time. In vitro treatment of DU145 cells with simvastatin induced HMG-CoA-reductase levels.

**Conclusion:**

Ionizing radiation more profoundly reduces proliferation as compared to simvastatin exposure, while the combined application of both modalities is synergistic. The inhibition of CoA-reductase may contribute to these effects.

## Introduction

Prostate cancer is the most common malignant neoplasia of men and a common cause of cancer-related death. It is predominantly a disease of older men, who frequently have the need for lipid-lowering therapy—predominantly statin therapy—to reduce cardiovascular risk.

Radiotherapy and surgery are the two mainstays of therapy with roughly identical therapeutic results on survival outcomes. Thus, many patients are exposed to radiotherapy as well as to statin medication.

The widespread use of statins in prostate cancer patients arose the question as to the combined effect of ionizing radiation and statin treatment on prostate cancer cells. This was addressed with a continuous cell culture model in vitro. Additionally, some experimental insight into the underlying mechanism of action ought to be generated.

Statins were reported to induce a growth arrest from G1- to S-phase of the cell cycle (Lee et al. 1998). Other studies showed the induction of apoptosis by statins (Wong et al. 2002). Previous studies also addressed the interaction of statins and ionizing radiation (He et al. 2012; Chen et al. 2018). However, these focused more on elucidating possible mechanisms of action of statins’ radiosensitizing activity as opposed to a more detailed characterisation of the effects of time of simvastatin exposure, radiation dose, time intervals and sequencing of modalities.

## Materials and methods

### Cell culture

DU145 prostate carcinoma cells (Stone et al. 1978) were grown in continuous cell culture in RPMI 1640 medium supplemented with 5 ml HEPES and 10% fetal bovine serum at 37° C in an incubator in the presence of 5% CO_2._ When subconfluent, cells were passaged to a new petri dish through trypsinization.

### Clonogenic assay

After detaching by trypsinization, the cell count was determined with a sceptre (Scepter 2.0 Handheld Automated Cell Counter, Merck). As needed, cells were then transferred to 6 cm petri dishes. They were then incubated mostly for 12 days and during this time radiation or drug treatment or both were applied as planned. After this, the medium was cautiously removed, the cells washed with PBS (phosphate buffered saline) and 1 ml crystal violet solution added. Resultant colonies were stained, while the dishes were gently moved on a shaker. The dishes were then shortly rinsed with water. Colonies were counted manually (Franken et al. 2006). A colony was defined as an agglomeration of at least 50 cells. Any measurements were done in triplicate, and results are shown as the mean of these estimates.

### Crystal violet assay

After the clonogenic assay, the dishes were subjected to a crystal violet assay (Ochel 2005). To the dishes, 1 ml of acetic acid was added and the dishes left on a shaker. After dissolution of crystal violet, three aliquots of 200 μl were transferred to a 96-well plate and the extinction measured at 540 nm in a fluorometer.

### Western blot

The western blot was done as reported (Ochel et al. 1999). Briefly, at the end of in vitro treatment, the cells were washed and lysed in TNES + PAL. After centrifugation the protein concentration of the supernatant was determined fluorometrically with a BCA-assay. Protein aliquots were then subjected to SDS-PAGE. The proteins were then blotted on activated PVDF membrane. After transfer and incubation with primary and secondary antibody, the chemiluminescent signal was obtained with the Supersignal system (Pierce).

## Results

(1) Radiation dose dependence

One hundred DU145 cells each were seeded in 16 six-centimetre petri dishes in medium containing 4 μM or 10 μM simvastatin or an equivalent quantity of DMSO solvent. Two days later half of the dishes were irradiated with 1–5 Gy (200 kV photons). Twelve days later the resultant colony numbers were determined followed by the crystal violet assay.

**Fig. 1:**
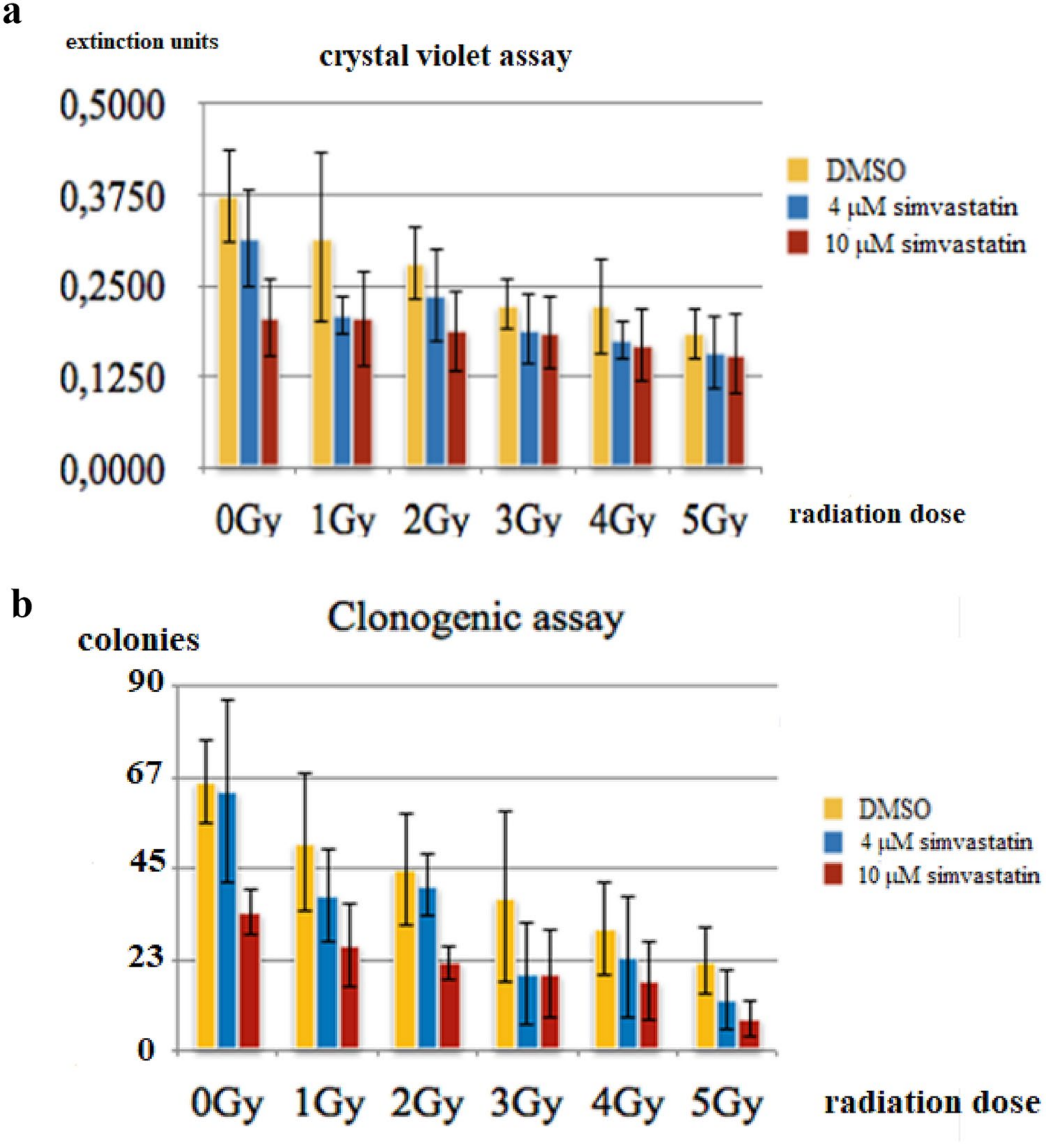
**a** Radiation dose dependence, crystal violet assay for colony formation after 12 days (means/standard deviations). **b** Radiation dose dependence, colony count after 12 days. (means/standard deviations)

A radiation dose dependence as well as a simvastatin drug dose–effect can be seen in both the clonogenic assay (Fig. 1a) and the crystal violet assay (Fig. 1b), although with considerable—but typical—experimental variation.

(2) Time of simvastatin addition

**Fig. 2:**
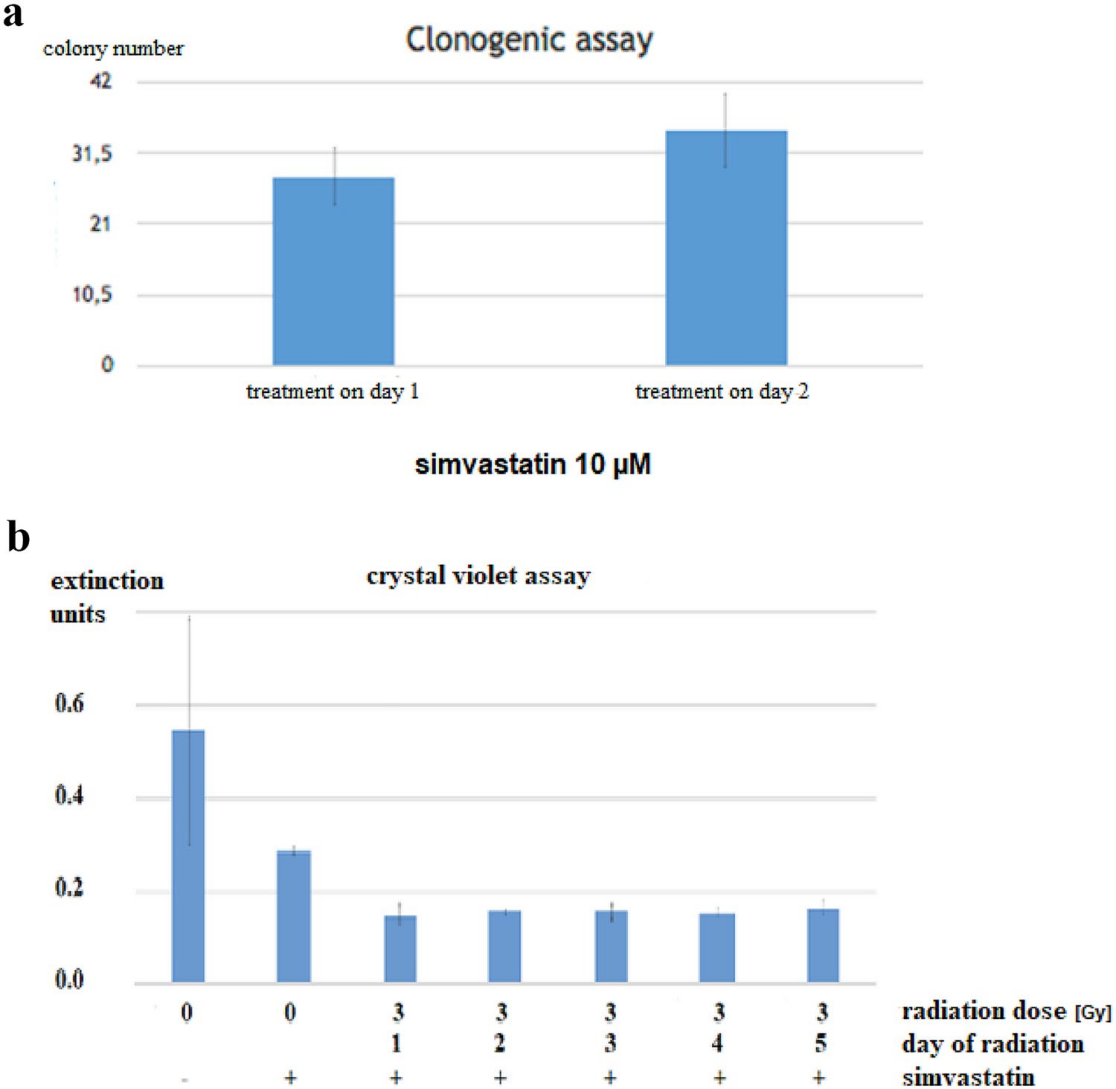
**a** Effect of simvastatin on colony formation depending on day of simvastatin addition. **b** Effect of 4 μM simvastatin plus radiation after different time-intervals from cell seeding, crystal violet assay, analysis after 13 days of cell culture. (means/standard deviations)

Next, the question was addressed if the exact time of simvastatin exposure influences the proliferation of DU145 cells. Therefore, simvastatin was added at the time of cell seeding into the dishes versus one day later. The colony count followed 13 days (immediate exposure) and 14 days (exposure one day later). The results are shown in Fig. 2a. The means are not significantly different (t-test).

(3) Significance of radiation timing

To elucidate the effect of the exact time point of radiation, 100 DU145 cells were seeded in 6 cm dishes. Dishes with either no treatment or exposed to 4 μM simvastatin only served as controls. As can be seen in Fig. 2a, b clear synergistic decrease of proliferation is exerted by the combined treatment with radiation and simvastatin in the crystal violet assay with almost identical results in the clonogenic assay (not shown).

(4) Simvastatin treatment for several days

**Fig. 3:**
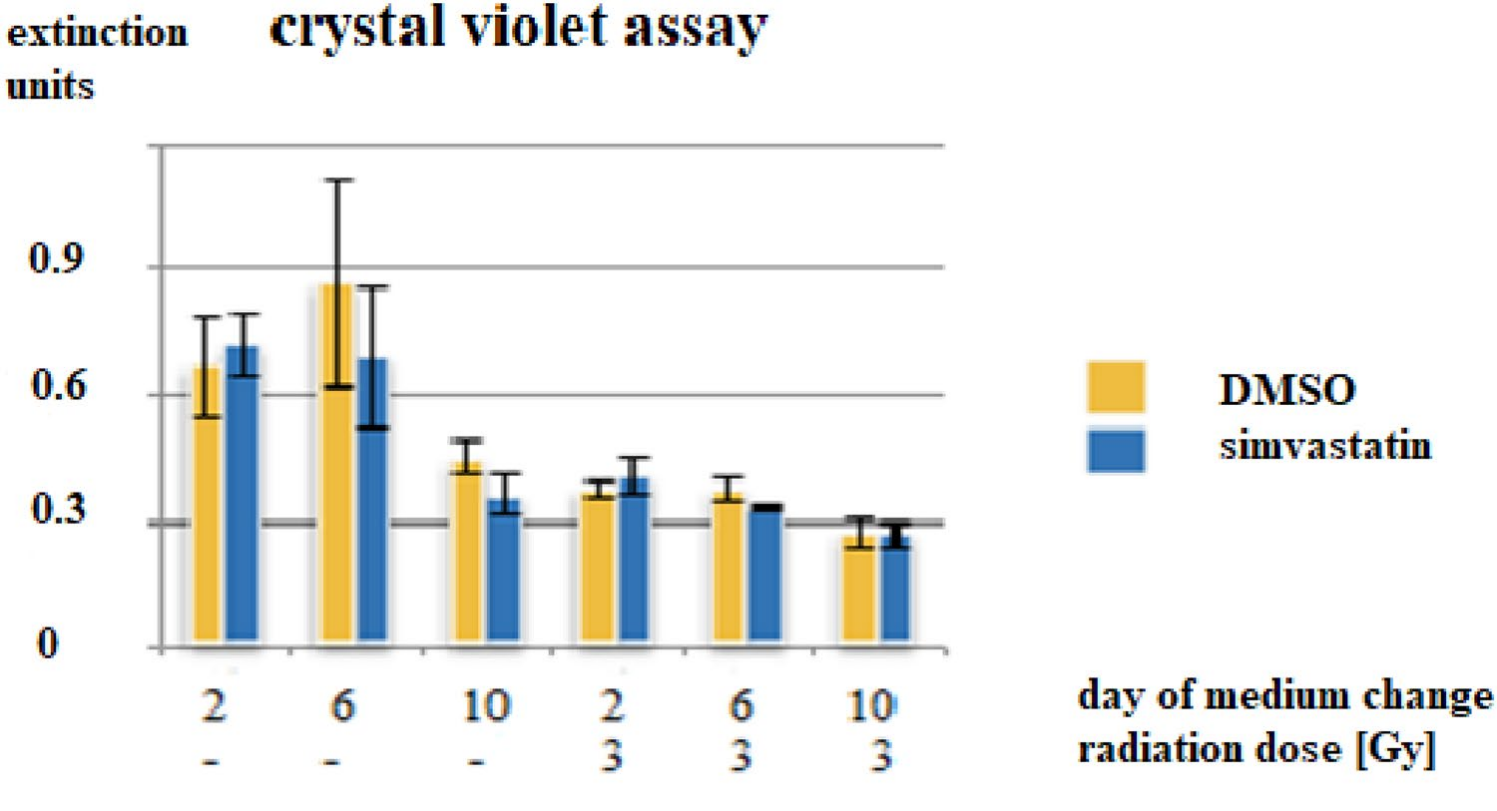
Crystal violet assay of DU145 cells exposed to different time-intervals of simvastatin treatment. They were irradiated and simvastatin was added, both one day after inoculation of DU145 cells. (means/standard deviations)

To examine if and to what extent the duration of simvastatin treatment influences DU145 prostate cancer cells, these were exposed to simvastatin for several days. Figure 3 depicts the results after 12 days of culture. The day after seeding, simvastatin was added and one half of the dishes were also irradiated. “Day of medium change” denotes the day when solvent DMSO- or simvastatin-containing medium was replaced by cell culture medium only.

(5) Mevalonolacton effect

**Fig. 4:**
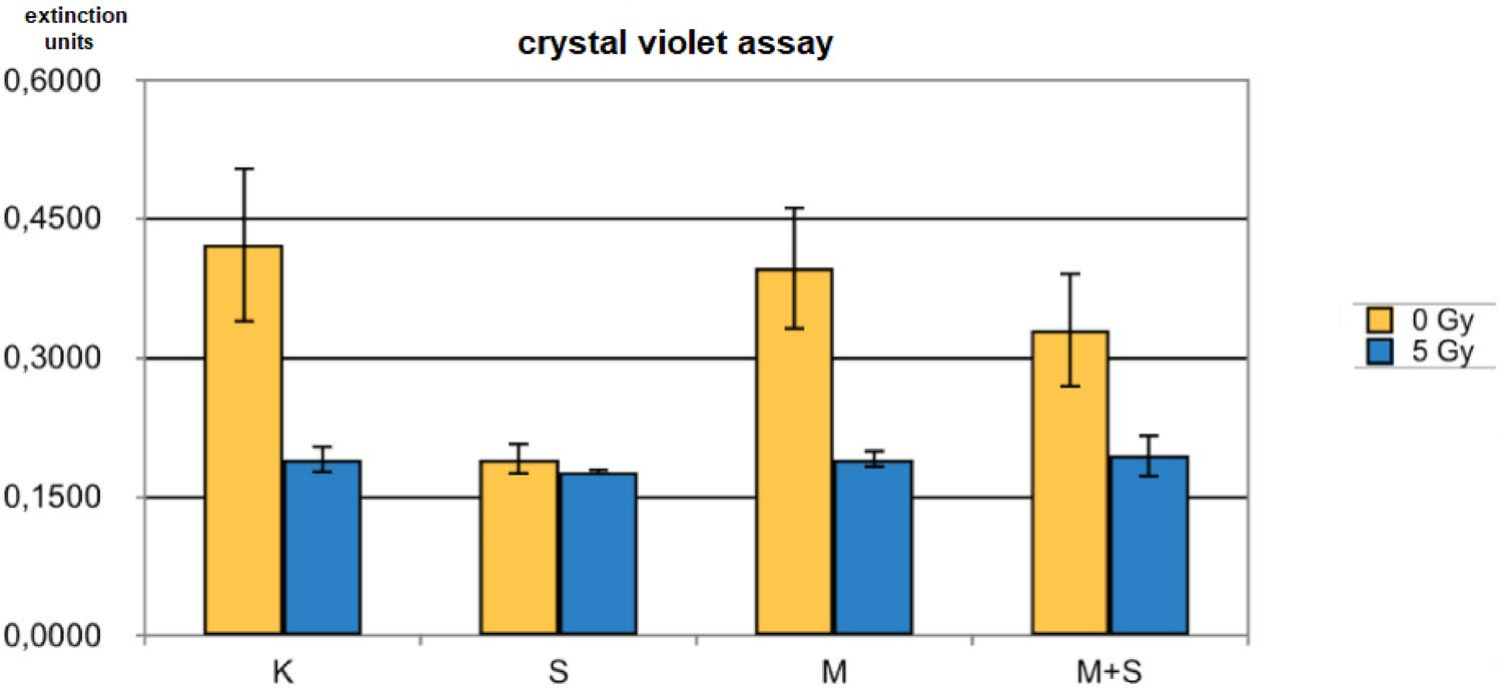
Crystal violet assay of colony formation of DU 145 cells after twelve days of treatment with solvent only, simvastatin, mevalonolacton or simvastatin combined with mevalonolacton (C: DMSO control, S: simvastatin 10 µM, M: mevalonolacton 2.5 mM; means/standard deviations)

Inhibition of the enzyme HMG-CoA-reductase is considered to be the mechanism of action for the lipid-lowering activity of simvastatin. The addition of mevalonlacton, the physiologic end-product of HMG-CoA-reductase enzymatic activity, should therefore counteract simvastatin's antiproliferative effect. As shown in Fig. 4 this was indeed, at least partially, observed.

(6) Effect of simvastatin acid

Since simvastatin is converted in the body to its active derivative simvastatin acid, the question arose in how far differences might exist as to the comparative in vitro activity of these compounds. Therefore, both were applied to DU145 cells under equivalent conditions. The crystal violet analysis shows comparable results on cellular proliferation. In 5 of the 6 conditions tested, the numerical extinction value for simvastatin acid is lower than that for simvastatin perhaps hinting towards a slightly higher antiproliferative activity of simvastatin acid. However, confidence intervals are broadly overlapping.

(7) Effect on HMG-CoA-reductase

**Fig. 5:**
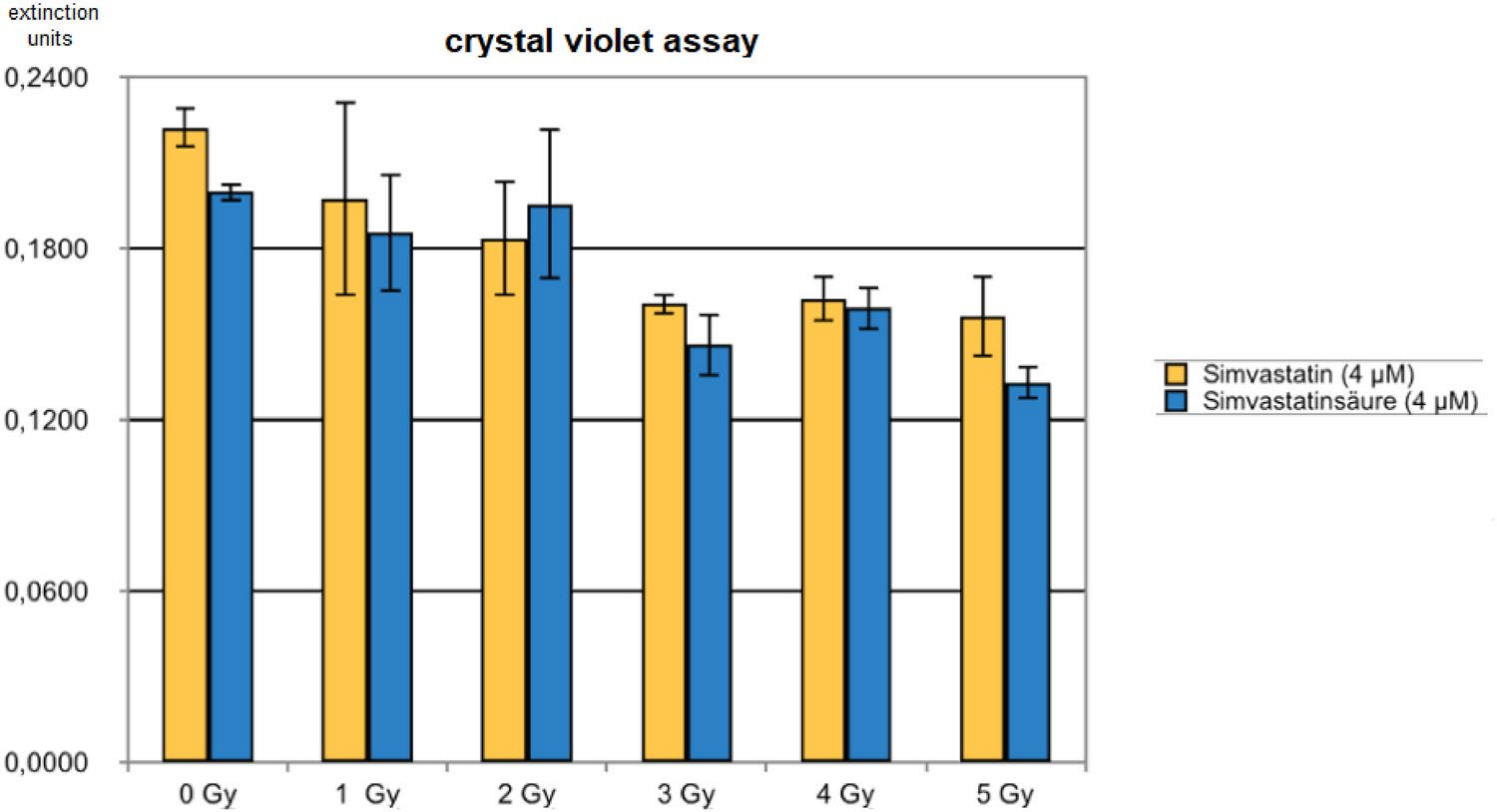
Comparative analysis of DU145 cells treated for 12 days with simvastatin or simvastatin acid. Radiation was applied one day after seeding. (means/standard deviations)

To further elucidate simvastatin's mode of action, DU145 cells were treated with medium or DMSO only as controls or with 4 μM or 10 μM simvastatin for 3 or 7 days and HMG-CoA-reductase levels analysed by western blot. As shown in Fig. 5, a time-dependent induction of this enzyme could be demonstrated. At both time points simvastatin at 3 μM gave a stronger signal than with treatment with 10 μM.

## Discussion

The use of statins concomitant to definitive radiotherapy of prostate cancer patients results in better biochemical recurrence-free survival compared to patients without this comedication during treatment (Gutt et al. 2010; Kollmeier et al. 2011). This was the incentive for the current investigation. Its focus is the in vitro reaction of prostate cancer cells to the combined effect of simvastatin and ionizing radiation. It could be demonstrated that both modalities impact the survival of DU145 prostate cancer cells in continuous cell culture, a cell line not yet evaluated in this setting. Contrary to previously published reports focusing more on elucidating the mechanism of action of simvastatin’s radiosensitizing quality, this report looks closely at the intricate details of the various experimental aspects of a radiosensitizing drug with radiation, especially aspects of radiation dose, timing and sequencing of modalities. This was shown in the colony formation assay as well as by means of the crystal violet assay. A dose and time dependence of cellular survival due to simvastatin treatment was obvious. These results are in line with those from experiments on cells from an adenocarcinoma of the lung showing a synergistic effect of ionizing radiation and lovastatin treatment (Sanli et al. 2011).

Efforts were undertaken to try to elucidate simvastatin's mechanism of action.

Since simvastatin is thought to act through inhibition of HMG-CoA-reductase (Goldstein and Brown 1990) and thereby decrease the concentration of mevalonolacton, a logical check is to add mevalonolacton to the experimental setup to see if simvastatin’s influence on cellular survival may be antagonized. This was indeed the case as depicted in Fig. 4. After additional radiation this effect was greatly diminished due to the more pronounced action of ionizing radiation. These results confirm the hypothesis that simvastatin’s cellular activity is at least partially mediated by HMG-CoA-reductase (Goldstein and Brown 1990).

Simvastatin is thought to act through its hydrolytic derivative simvastatin acid (Alberts 1990). The comparative activity of these compounds was tested and yielded almost identical results, supporting, but not proving, the claim that simvastatin acid is the active principle behind simvastatin’s cellular effects.

Another possible consequence of simvastatin treatment might be changes in the ubiquitin–proteasome system. Investigating statin-induced myopathy, Urso et al (2005) showed increased expression of proteins involved in the ubiquitin/proteasome pathway (UPP) in muscle biopsies after statin treatment and physical exercise. Therefore, searching for changes in the UPP system, DU145 cells were treated with DMSO as a solvent control, with 4 μM simvastatin or 10 μM simvastatin over 24 h. After that, cells were lysed and a western blot analysis of whole protein lysates for ubiquitin was done. There were no discernible differences between the typically elongated western blot signals from either treatment condition. This result rules out a global, negative impact on cellular ubiquitination, but cannot exclude the possibility that some defined proteins exert changes in ubiquitination. Subtle changes from a small, but potentially important minority of proteins may thus still exist.

**Fig. 6:**
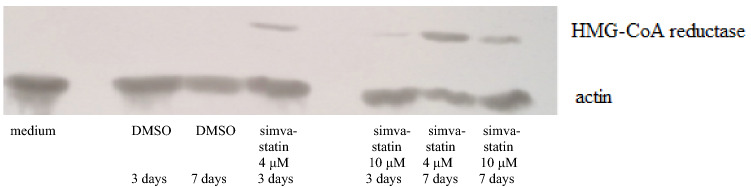
Time-dependent induction of HMG-CoA-reductase by simvastatin

Finally, going back to the assumption, that inhibition of HMG-CoA-reductase is of major importance for simvastatin’s cellular survival effects, a western blot analysis was undertaken (Fig. 6). In the absence of simvastatin (DMSO solvent control), no signal for this enzyme could be seen, while treatment with 4 μM simvastatin clearly induced it. The signal for HMG-CoA-reductase after 10 μM simvastatin was weaker than after treatment with 4 μM, perhaps due to increased toxicity or enzyme degradation.

One possible, mechanism behind these results was described by Park et al (2001). He found that DU145 prostate carcinoma cells treated with lovastatin expressed less E2F-1 transcription factor, resulting in increased apoptosis and a reduction of the number of cells in the S-phase of the cell cycle.

Further insights into the mechanism of action of simvastatin’s radiosensitizing effect stem from a publication from Zhenhua et al. (He et al. 2012). They found that the activation of the autophagy system by simvastatin induces apoptosis and may thus confer an increased radiosensitivity.

Chen et al. through the use of comet assays provided evidence that simvastatin inhibits the repair of double-strand breaks resulting in increased apoptosis and synergy with radiation-induced cell death (Chen et al. 2018).

In summary, the experiments presented here confirm that in prostate cancer cells simvastatin has a time- and dose-dependent radiation-sensitizing effect. In the case of simvastatin, this seems to be conferred through its active derivative simvastatin acid and is sensible to mevalonolacton antagonization. The results described herein provide a preclinical basis for the above mentioned favourable clinical outcomes for prostate cancer patients on statin therapy while receiving radiotherapy. Alterations in HMG-CoA-reductase levels strongly suggest that—as in other biological systems—this enzyme plays a major role in simvastatin’s cellular survival effects on prostate cancer cells.

## Data Availability

The datasets used and analysed during the current study are available from the corresponding author on reasonable request.
